# Supplementary material for: Stereotactic Body Radiotherapy (SBRT) to Localised Prostate Cancer in the Era of MRI-Guided Adaptive Radiotherapy: Doses Delivered in the HERMES Trial Comparing Two- and Five-Fraction Treatments
Source: Cancers (Basel). 2024 May 30;16(11):2073. doi: 10.3390/cancers16112073 (PMC11171331; doi:10.3390/cancers16112073)
Supplement: Supplementary file 1 [file cancers-16-02073-s001.zip › cancers-3010963-supplementary.pdf]

Supplementary material

Supplementary material S1a

Dose constraints used in the planning of the 2-fraction regimen.

	Planning Dosimetric Criterion		
**Structure**	**Dose Metric**	**Optimal**	**Mandatory**
**GTV**	V27 Gy	> 95%	> 90%
	D0.1 cm^3	-	< 33.75 Gy
**PTV1**	V24 Gy	> 95%	> 75%
	D98%	> 22.8 Gy	> 17 Gy
**PTV2**	V20 Gy	> 95%	> 75%
**Urethra**	D10%	< 26 Gy	< 27 Gy
**Bowel**	V12 Gy	-	< 5 cm^3
**Femoral head_L**	V14 Gy	< 10 cm^3	
**Femoral head_R**	V14 Gy	< 10 cm^3	
**Bladder**	V20.8 Gy	-	< 5 cm^3
	V14.6 Gy	-	< 15 cm^3
**Penile Bulb**	V19.5 Gy	< 50%	< 100%
**Rectum**	V20.8 Gy	-	< 1 cm^3
	V17.6 Gy	-	< 4 cm^3
	V13 Gy	< 7 cm^3	

Supplementary material S1b

Dose constraints used in the planning of the 5-fraction regimen (based on PACE B protocol).

	Planning Dosimetric Criterion		
**Structure**	**Dose Metric**	**Optimal**	**Mandatory**
**CTV1**	V40 Gy	-	> 95%
**PTV1**	D0.1 cm^3	-	< 48 Gy
	V36.25 Gy	-	> 95%
	D98%	34.4 Gy	33.72 Gy
**PTV2**	V30 Gy	-	95%
**Urethra**	V42 Gy	< 50%	
**Bowel**	V30 Gy	< 1 cm^3	< 2 cm^3
	V18.10 Gy	-	< 5 cm^3
**Femoral head_L**	V14.50 Gy	< 5%	
**Femoral head_R**	V14.50 Gy	< 5%	
**Bladder**	V37 Gy	<5 cm^3	10 cm^3
	V18.10 Gy	-	< 40%
**PenileBulb**	V29.50 Gy	< 50%	
**Rectum**	V36 Gy	< 1 cm^3	< 2 cm^3
	V29 Gy	-	< 20%
	V18.1 Gy	-	< 50%

Supplementary material S2.

The dose to the targets in the 2-fraction cohort

		CTVpsv			CTVpsv			CTVpsv			CTVsv			CTVsv			CTVsv			GTVpb_2700			GTVpb_2700		
		V2400cGy			D95%			D98%			V2000cGy			D95%			D98%			V2700cGy			V2700cGy > 95% (-5%)		
Patient	subfraction	Session	Verif	Post	Session	Verif	Post	Session	Verif	Post	Session	Verif	Post	Session	Verif	Post	Session	Verif	Post	Session	Verif	Post	Session	Verif	Post
1	1.1	98.1	96.9	99.6	24.5	24.3	24.3	24.0	23.7	23.5	100.0	99.8	98.2	23.8	23.2	24.3	22.8	22.1	23.5	99.5	99.5	99.4	27.7	27.7	27.7
1	1.2	98.5	98.7	98.6	24.5	24.6	24.5	24.1	24.2	24.2	100.0	100.0	98.6	23.8	23.8	23.3	22.9	22.5	22.1	99.9	100.0	97.7	27.6	27.8	27.3
1	2.1	99.5	97.6	97.0	25.0	24.8	24.7	24.6	23.7	23.1	100.0	100.0	99.7	24.3	23.2	22.9	23.3	22.3	21.9	100.0	100.0	99.8	27.8	27.8	27.7
1	2.2	98.7	98.0	98.8	24.7	24.6	24.7	24.2	24.0	24.3	100.0	100.0	100.0	24.0	23.9	24.1	23.1	23.0	24.3	100.0	96.2	96.0	28.1	27.1	27.0
2	1.1	97.1	91.5	76.0	24.4	23.0	16.8	23.7	21.6	14.9	100.0	97.5	81.1	24.4	21.6	11.0	23.7	19.1	7.1	97.3	97.6	87.5	27.7	27.2	26.7
2	1.2	96.6	95.6	92.2	24.3	24.1	23.2	23.6	23.2	21.8	100.0	99.7	96.9	23.8	23.3	21.3	23.0	22.2	18.7	96.1	94.9	87.1	27.1	27.0	26.4
2	2.1	96.4	93.1	92.0	24.4	23.4	22.9	23.3	21.5	21.0	100.0	99.0	98.2	23.6	22.1	21.3	22.8	20.9	20.1	97.9	85.7	95.7	27.3	26.4	27.1
2	2.2	98.2	98.3	94.2	24.6	24.7	21.7	24.1	24.1	23.8	100.0	100.0	99.1	24.3	24.2	23.0	23.6	23.2	21.3	97.3	97.5	98.7	27.2	27.3	27.4
3	1.1	98.5	99.8	99.8	24.9	25.1	25.1	24.4	24.8	24.9				0.0	0.0	0.0	0.0	0.0	0.0	98.6	100.0	98.9	28.0	28.6	28.0
3	1.2	97.7	99.6	95.4	24.7	25.3	24.1	23.9	24.8	22.4				0.0	0.0	0.0	0.0	0.0	0.0	99.1	99.2	99.6	27.9	26.3	27.0
3	2.1	98.3	99.4	99.4	24.6	24.8	24.8	24.1	24.4	24.4				0.0	0.0	0.0	0.0	0.0	0.0	99.5	86.8	98.8	28.6	26.4	27.6
3	2.2	96	96	91	24.2	24.4	22.8	23.2	23.4	21.1				0.0	0.0	0.0	0.0	0.0	0.0				27.7	24.4	28.2
4	1.1	100.0	99.9	99.9	24.8	24.9	24.8	24.6	24.6	24.5	100.0	100.0	100.0	24.5	24.5	24.4	23.4	23.3	23.3	97.2	97.0	98.1	27.3	27.3	27.5
4	1.2	100.0	99.8	99.9	25.1	25.0	25.0	24.8	24.7	24.7	100.0	100.0	100.0	24.6	24.8	24.6	23.9	24.2	24.0	98.6	98.1	93.3	27.4	27.3	26.9
4	2.1	99.9	99.4	99.7	25.2	25.2	25.2	24.9	24.7	24.9	100.0	100.0	100.0	24.7	24.6	24.7	23.7	23.5	23.7	99.9	100.0	95.9	27.7	28.0	27.1
4	2.2	99.7	98.9	99.5	24.9	24.9	24.8	24.6	24.4	24.5	100.0	100.0	100.0	24.3	24.3	24.2	23.1	23.4	23.2	98.7	98.2	98.2	27.3	27.2	27.3
5	1.1	91.4	92.1	92.5	23.1	23.4	23.4	21.6	22.1	21.8				0.0	0.0	0.0	0.0	0.0	0.0	99.3	100.0	99.9	27.7	27.8	27.9
5	1.2	93.5	90.7	87.5	23.7	22.9	21.9	22.5	21.6	20.1				0.0	0.0	0.0	0.0	0.0	0.0	98.1	99.2	96.3	27.4	27.6	27.2
5	2.1	98.7	96.9	98.6	25.5	24.4	20.4	24.2	23.4	24.2				0.0	0.0	0.0	0.0	0.0	0.0	99.8	98.2	98.6	27.7	27.3	27.3
5	2.2	97.5	95.4	93.7	24.4	24.1	23.8	23.9	22.9	22.7				0.0	0.0	0.0	0.0	0.0	0.0	99.0	98.8	97.1	27.3	27.3	27.2

Supplementary material S3.

The dose to the targets in the 5-fraction cohort

		CTVpsv			CTVpsv			CTVpsv			CTVpsv			CTVpsv			CTVsv			CTVsv			CTVsv		
		V4000cGy > 95%			D95% > 4000 cGy			V3625 cGy > 95%			D95% > 3625Gcy			D98% > 3625 cGy			V3000 cGy > 95%			D95% > 3000 cGy			D98% > 3000 cGy		
Patient no.	fraction	Session	Verif	Post	Session	Verif	Post	Session	Verif	Post	Session	Verif	Post	Session	Verif	Post	Session	Verif	Post	Session	Verif	Post	Session	Verif	Post
1	1	94.7	96.6	90.8	39.9	40.4	38.6	100.0	100.0	98.4	39.9	40.4	38.6	39.0	39.3	36.6	100.0	99.8	100.0	37.6	36.4	36.3	35.5	33.4	34.3
1	2	95.8	92.5	91.7	40.0	39.3	38.9	100.0	99.7	98.7	40.0	39.3	38.9	39.1	38.0	37.0	100.0	100.0	100.0	27.6	36.8	36.3	35.1	34.8	34.5
1	3	98.2	87.5	85.9	40.7	37.5	36.6	100.0	97.0	95.7	40.7	37.5	36.6	40.1	35.5	34.5	100.0	99.8	99.7	37.3	36.1	35.4	34.9	34.1	33.2
1	4	96.9	93.3	92.2	40.3	39.5	38.8	100.0	99.6	98.7	40.3	39.5	38.8	39.7	38.0	36.9	100.0	100.0	99.8	37.0	37.2	36.1	34.4	34.8	34.0
1	5	96.0	97.8	44.9	40.2	40.7	20.8	100.0	100.0	58.0	40.2	40.7	20.8	39.5	39.9	18.5	100.0	99.9	71.6	38.0	38.1	20.2	35.1	35.2	17.8
2	1	95.8	95.0	83.3	40.1	40.0	36.7	100.0	100.0	96.1	40.1	40.0	36.7	39.3	39.2	35.1	100.0	99.9	100.0	36.2	37.0	35.3	34.3	34.7	34.1
2	2	96.2	96.9	96.9	40.3	40.4	40.4	100.0	100.0	100.0	40.3	40.4	40.4	39.4	39.7	39.6	100.0	100.0	99.7	37.3	37.8	37.3	35.0	35.4	34.7
2	3	95.5	88.6	66.8	40.1	38.0	30.7	100.0	98.3	82.7	40.1	38.0	30.7	39.3	36.4	28.1	100.0	100.0	95.9	37.6	36.3	30.5	36.0	35.2	28.3
2	4	95.1	96.4		40.0	40.2	0.0	100.0	100.0		40.0	40.2	0.0	39.2	39.4	0.0	100.0	100.0		37.5	38.1	0.0	35.5	36.2	0.0
2	5	95.4	89.8	72.1	40.1	38.8	33.0	100.0	99.5	87.3	40.1	38.8	33.0	39.2	37.6	31.0	100.0	100.0	98.2	37.0	36.4	32.0	35.3	34.7	30.1
3	1	94.1	91.1	90.3	39.8	39.3	38.8	100.0	99.5	98.2	39.8	39.3	38.8	39.0	38.1	36.6	100.0	98.2	96.0	35.6	34.7	31.6	34.0	30.4	26.9
3	2	93.8	87.8	88.1	39.8	38.1	37.8	100.0	98.0	96.8	39.8	38.1	37.8	39.1	36.2	33.9	100.0	98.9	93.2	35.2	33.9	27.3	33.7	31.4	22.7
3	3	94.8	94.5	93.0	40.0	39.9	39.7	100.0	100.0	100.0	40.0	39.9	39.7	39.5	39.4	39.0	100.0	100.0	100.0	35.8	36.2	38.4	33.8	34.2	36.1
3	4	96.4	96.5	86.9	40.3	40.4	38.3	100.0	99.8	98.8	40.3	40.4	38.3	39.4	39.4	37.0	100.0	100.0	100.0	35.7	39.4	36.9	34.3	37.6	35.1
3	5	95.8	90.8	84.3	40.1	39.2	38.1	100.0	99.6	99.0	40.1	39.2	38.1	39.4	38.0	37.0	100.0	100.0	100.0	35.5	36.7	36.9	33.9	34.7	35.5
4	1	97.6	95.9	93.3	40.4	40.2	39.6	100.0	99.9	99.8	40.4	40.2	39.6	39.9	39.5	38.4	100.0	100.0	100.0						
4	2	96.4	93.4	94.3	40.2	39.7	39.9	100.0	100.0	100.0	40.2	39.7	39.9	39.7	39.0	39.2	100.0	100.0	100.0						
4	3	96.9	94.5	94.8	40.3	39.9	40.0	100.0	100.0	100.0	40.3	39.9	40.0	39.8	39.1	39.2	100.0	100.0	100.0						
4	4	95.1	94.1	92.2	40.0	39.9	39.5	100.0	100.0	99.7	40.0	39.9	39.5	39.6	39.2	38.4	100.0	100.0	100.0						
4	5	95.7	93.9	94.9	40.1	39.8	40.0	100.0	100.0	100.0	40.1	39.8	40.0	39.5	38.9	39.2	100.0	100.0	100.0						
5	1	96.2	91.1	84.1	40.2	39.0	36.6	100.0	98.8	95.6	40.2	39.0	36.6	39.6	37.4	34.3	100.0	100.0	99.7						
5	2	96.1	94.6	92.3	40.1	39.9	39.3	100.0	100.0	99.5	40.1	39.9	39.3	39.6	38.9	38.0	100.0	100.0	100.0						
5	3	97.5	95.9	87.3	40.4	40.1	38.2	100.0	100.0	97.9	40.4	40.1	38.2	39.9	39.5	36.2	100.0	100.0	100.0						
5	4	96.3	96.5	98.0	40.2	40.3	40.5	100.0	100.0	100.0	40.2	40.3	40.5	39.6	39.7	40.0	100.0	100.0	100.0						
5	5	95.0	92.7	89.6	40.0	39.7	39.3	100.0	99.8	99.9	40.0	39.7	39.3	39.5	38.9	38.5	100.0	100.0	100.0						

Supplementary material S4.

The dose to the OARs in the 2-fraction cohort

		Urethra			Bladder			Bladder			Rectum			Rectum		
		D10% <26 Gy (+1 Gy)			D5 cm3 < V20.8 Gy			D15 cm3 < V14.6 Gy			D1cm^3 < 20.8 Gy			D4cm3 < 17.6 Gy		
Patient no.	subfraction	Session	Verif	Post	Session	Verif	Post	Session	Verif	Post	Session	Verif	Post	Session	Verif	Post
1	1.1	26.3	26.3	26.3	17.2	21.9	22.4	11.4	15.8	16.7	20.6	21.2	21.5	17.4	17.1	16.6
1	1.2	25.9	25.9	25.7	17.7	19.6	17.6	11.9	13.2	11.6	20.7	21.3	23.4	16.4	16.4	18.8
1	2.1	26.5	26.6	26.7	18.5	18.1	15.9	12.4	11.6	10.4	21.6	22.1	23.7	15.5	16.5	18.3
1	2.2	26.2	26.1	26.1	18.2	19.5	20.0	12.2	13.2	13.7	21.2	19.9	19.2	15.3	14.1	13.7
2	1.1	25.8	26.2	26.5	19.9	17.0	11.3	13.5	11.6	7.7	20.3	21.2	25.7	15.5	15.7	23.4
2	1.2	26.0	25.8	25.9	20.2	19.1	16.7	14.4	13.2	11.5	20.8	20.8	24.4	16.8	16.8	21.3
2	2.1	26.4	26.2	26.3	20.0	17.0	15.6	14.2	12.1	11.2	20.7	23.4	25.7	16.6	19.3	22.7
2	2.2	26.2	26.0	26.4	19.9	17.8	14.9	14.0	12.7	10.9	20.5	22.9	25.3	15.9	18.0	21.1
3	1.1	26.0	26.5	26.2	18.8	20.9	22.7	11.9	13.6	15.2	20.1	22.1	22.5	14.0	15.9	16.2
3	1.2	25.9	26.3	26.3	19.3	20.8	19.2	12.8	14.4	12.7	20.6	22.3	26.5	14.8	17.0	22.6
3	2.1	25.9	26.1	26.2	20.2	20.3	18.3	13.0	12.7	11.3	20.4	22.4	23.8	14.5	16.9	18.8
3	2.2	25.7	25.7	26.2	20.1	19.9	21.2	13.8	13.4	15.1	21.2	21.1	26.6	15.8	14.2	24.0
4	1.1	25.9	26.1	26.1	20.8	22.3	23.0	12.6	15.6	16.6	19.2	18.2	18.1	14.3	13.6	13.3
4	1.2	26.8	26.4	26.9	19.2	21.0	23.9	13.7	14.5	17.8	18.9	18.1	16.0	13.7	14.0	12.7
4	2.1	26.9	26.9	26.3	19.5	19.0	20.5	14.2	13.9	14.6	17.9	16.3	16.4	13.4	13.1	13.1
4	2.2	26.7	26.9	26.5	19.6	19.6	21.1	14.2	13.8	15.3	18.4	17.1	17.1	13.6	13.3	13.7
5	1.1	26.8	26.6	26.7	18.5	18.2	24.2	14.4	13.8	18.8	19.9	20.2	19.0	16.5	16.7	15.7
5	1.2	25.5	25.7	25.3	19.2	20.4	22.4	14.4	14.9	17.0	20.5	19.0	16.6	17.2	15.5	13.4
5	2.1	26.5	25.5	26.7	17.9	18.8	21.0	14.0	14.8	16.6	20.5	20.2	20.5	15.9	15.5	15.8
5	2.2	26.0	26.2	26.1	18.4	18.1	18.8	14.2	14.0	14.8	20.6	22.9	22.6	16.1	18.3	17.8

Supplementary material S5.

The dose to the OARs in the 5-fraction cohort

With out air		Urethra			Bladder			Bladder			Rectum			Rectum		
		D50% < 4200 cGy			D5 cm3 < V3700 cGy			D40% < 1810 cGy			D1cm^3 < 3600 cGy			D20% < 2900 cGy		
Patient no.	subfraction	Session	Verif	Post	Session	Verif	Post	Session	Verif	Post	Session	Verif	Post	Session	Verif	Post
1	1	4210	4238	4228	3293.3	3885.3	4175.1	908.5	1006.1	1060.2	3603.9	3589.4	3348.4	2054.2	1983.9	1926.1
1	2	4103	4156	4135	2337.3	3630.9	3998.7	1114.6	1078.6	1096	3515.1	3331.7	3071.2	2214.9	2046.9	1997.4
1	3	4160	4170	4203	3543.5	3849.9	3935.9	1157.1	1236.3	1201.7	3633.5	2883.2	2815.1	2103.8	1764.4	1739.8
1	4	4170	4204	4179	3351.9	3643.9	3734.8	962.7	996.3	964.9	3534.2	3227.1	3044	2101.2	1889.1	1830.7
1	5	4242	4225	4274	3552.6	3617.2	1822.9	1190.6	1029	660.9	3570.3	3759.6	4501.7	2252.7	2330.3	4263.7
2	1	4184	4172	4172	3384.3	3382.4	3887.4	590.3	546.9	474.8	3581.9	3643.9	3154.2	2233.8	2550.7	1990.1
2	2	4169	4139	4143	3302.1	3217.9	3260.8	653.4	585.3	403.3	3654.2	3669.7	3739.5	2235.8	2378.1	2402.1
2	3	4200	4231	4246	3618.6	3444.4	4070.8	967.3	751.8	836.3	3614.3	3223.5	2506.7	2151.5	1901.7	1477
2	4	4166	4144		3453.7	3552.1		712.6	482.7		3689.7	3767.4		2428.8	2699.2	
2	5	4172	4166	4195	2527.3	3578.9	4117	858	541.2	992.2	3562.7	3339.1	2554.2	2113.8	1903.3	1504.3
3	1	4187	4237	4214	2952.4	2943.7	3072.5	615.3	549.3	576	3637.5	4152.5	4201.8	1895.3	2700.8	2926.4
3	2	4149	4158	4178	3062.6	3892.4	2852.2	565.3	713.6	275.5	3761.5	3266.7	4298.2	2025.7	1631.3	2868
3	3	4111	4104	4102	3342.3	3549.5	3489.6	586.9	620.4	577.8	3601.5	3575.3	3814.8	1912.6	1878.4	2243.2
3	4	4189	4243	4225	3535.2	3843.2	3024.8	644.2	714.9	422.7	3628.8	3729	4185.6	1899.5	1975	2845.9
3	5	4166	4204	4134	3362.6	3884.6	3849.1	684.5	795.1	844.1	3630.2	3299.7	3455.7	1845.8	1677.8	1884.1
4	1	4178	4171	4125	3041.5	2980.3	2872.3	449.2	393.3	379	3512.8	3685.8	3968	1439	1581.3	1752.1
4	2	4062	4061	4050	2977.2	3193.5	3212.1	340.4	356.2	359.4	3592.2	3357.8	3351.3	1505.1	1427.5	1446.6
4	3	4101	4133	4183	3265.1	2919.4	3288.2	509.6	376.6	487.4	3603.1	3844.5	3644.8	1450	1632	1483.3
4	4	4062	4072	4068	2831.5	2829.2	2722.5	420.8	431.3	382.3	3569.3	3138.5	3392.5	197.5	1353.6	1478.7
4	5	3959	4149	4127	3105.3	2753	2746.2	391.6	365.1	436.4	3563.7	3370.3	3421.5	1519.2	1511.4	1492.6
5	1	4100	4120	4112	2838.1	2665.8	1916	987.2	973.4	611.1	3179.2	3227.8	3943.3	1299.7	1322.2	1650.5
5	2	4116	4170	4198	3308.5	2647.5	2275.8	1430.5	900.1	797	3339.2	3480.5	3659.5	1326.1	1367.2	1458.7
5	3	4131	4158	4161	2659.7	2682.9	2055.5	963	902	546.8	3197.7	3209	3804.2	1391.4	1500.5	1677.6
5	4	4117	4140	4133	3064.2	2780.1	2906.3	1327.8	1029.4	701	3454	3154.3	3093.8	1397.2	1362.7	1350.1
5	5	4124	4110	4091	3039.5	2700.3	2415.7	1060.7	729.9	733.5	3392.2	3427.5	3453.2	1222.5	1303.1	1297.1

Supplementary material S6.

S6a.

Linear correlation coefficient between the difference in the rectal D1cm^3 (cGy) when calculated with and without the presence of air and the volume of air in the rectum within 1cm of the prostate volume in the 2-fraction cohort.

$R^2 = 0.67$

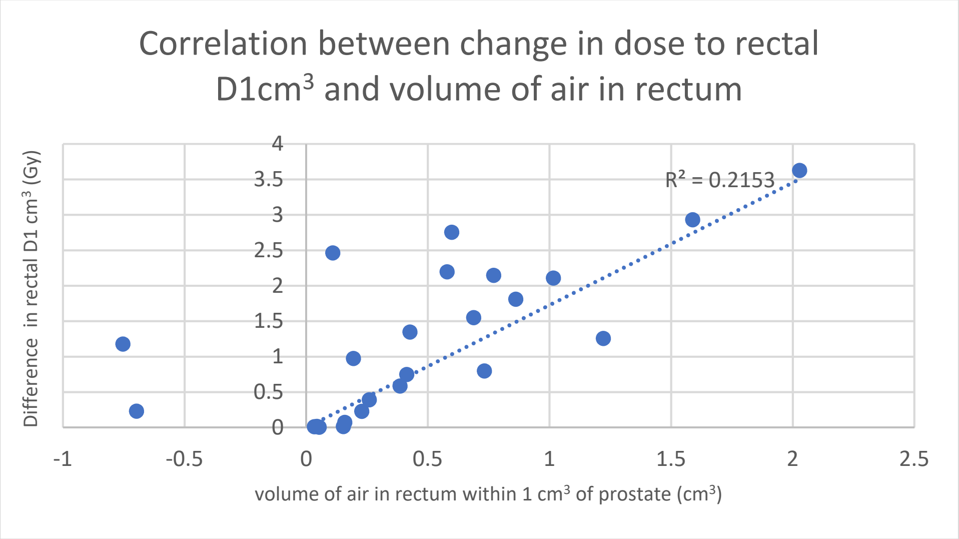

Supplementary material S6.

S6b.

Linear correlation coefficient between the difference in the rectal D1cm^3 (cGy) when calculated with and without the presence of air and the volume of air in the rectum within 1cm of the prostate volume in the 5-fraction cohort.

$R^2 = 0.91$

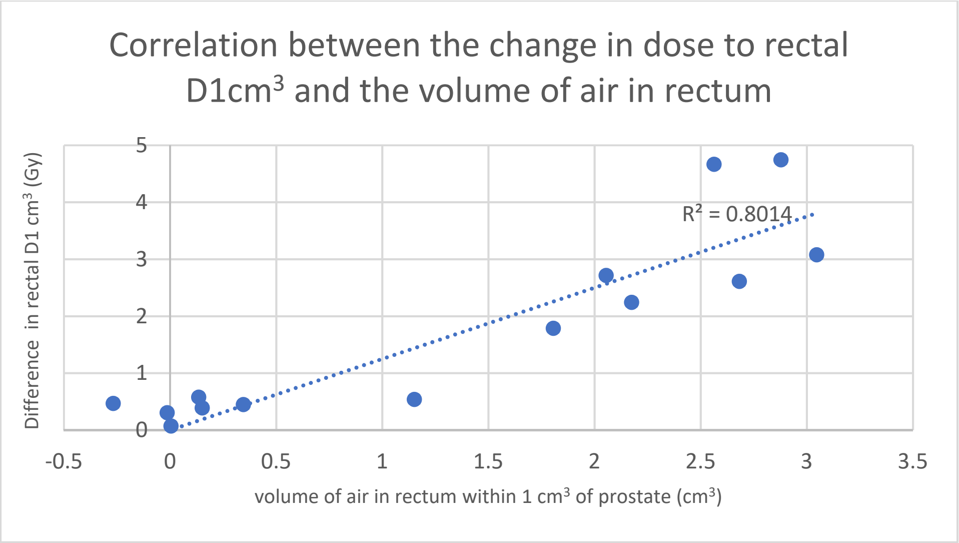

Supplementary material S7. S7a

Change is rectal D1 cm^3 when calculated with and without the presence of air in the in the 2-fraction cohort.

	Rectal D1cm^3 (Gy)					
	MRI_{verif}			MRI_{post}		
Patient	without air	with air	difference	without air	with air	difference
1	21.1	21.4	0.3	21.9	22.4	0.5
2	22.1	22.9	0.8	25.3	25.9	0.6
3	22.0	21.8	-0.2	24.8	24.7	-0.2
4	17.4	17.8	0.3	16.9	17.2	0.3
5	20.6	20.8	0.2	19.7	20.0	0.4
Cohort	21.1	21.4	0.3	21.9	22.4	0.5

Supplementary material S7. S7b

Change is rectal D1 cm^3 when calculated with and without the presence of air in the in the 2-fraction cohort.

	Rectal D1cm^3 (Gy)					
	MRI_{verif}			MRI_{post}		
Patient	without air	with air	difference	without air	with air	difference
1	33.6	33.6	0.0	33.6	33.9	0.4
2	35.3	35.3	0.0	29.9	29.8	-0.1
3	36.0	36.6	0.5	39.9	40.7	0.8
4	34.8	36.0	1.2	35.6	36.4	0.9
5	33.0	33.1	0.1	35.9	35.9	0.0
Cohort	34.8	35.3	0.5	35.6	35.9	0.4
